# Tantalum-specific contrast-to-noise ratio or conventional detector dose-driven exposure control in angiography: radiation dose and image quality evaluation in a porcine model

**DOI:** 10.1186/s41747-022-00275-z

**Published:** 2022-05-17

**Authors:** Thomas Werncke, Timo Christian Meine, Jan B. Hinrichs, Sabine K. Maschke, Lena Sophie Becker, Inga Brüsch, Regina Rumpel, Frank K. Wacker, Bernhard C. Meyer

**Affiliations:** 1 Department of Diagnostic and Interventional Radiology, Hannover Medical School, Carl-Neuberg-Str. 1, 30625 Hannover, Germany; 2 Institute for Laboratory Animal Science and Central Animal Facility, Hannover Medical School, Carl-Neuberg-Str. 1, 30625 Hannover, Germany

**Keywords:** Angiography (digital subtraction), Animals, Fluoroscopy, Radiation dosage, Tantalum

## Abstract

**Background:**

The aim of this animal study was to compare the fluoroscopic image quality (IQ) and radiation dose between a tantalum (Ta)-specific contrast-to-noise ratio-driven exposure control (Ta-CEC) and a detector dose-driven exposure control (DEC) in abdominal angiography.

**Methods:**

Nine angiography scenarios were created in seven anaesthetised pigs using Ta-based embolisation material during percutaneous liver and kidney intervention. Fluoroscopic images were acquired using three DEC protocols with different dose levels and Ta-CEC protocols with different IQ levels, sampled in small steps. Polymethyl-methacrylate and aluminium plates were used to simulate attenuation of three water equivalent thicknesses (WET). Three blinded readers evaluated the IQ of DEC and dose equivalent Ta images and selected the Ta-IQ equivalent image corresponding to the DEC image.

**Results:**

Interobserver agreement for the IQ assessment was 0.43 for DEC, 0.56 for Ta-CEC and for the assessment of incident air kerma at the interventional reference point (*K*_a,r_) for the Ta-IQ equivalent image 0.73. The average IQ of the dose equivalent Ta images was superior compared to the DEC images (*p* < 0.001) and also for every WET (26, 31, or 36 cm) and dose level (*p* ≤ 0.022). The average *K*_a,r_ for the Ta-IQ equivalent images was 59 ± 16% (mean ± standard deviation) lower compared to the DEC images (*p* < 0.001).

**Conclusions:**

Compared to DEC, Ta-CEC significantly improved the fluoroscopic depiction of Ta, while maintaining the *K*_a,r_. Alternatively, the *K*_a,r_ can be significantly reduced by using Ta-CEC instead of DEC, while maintaining equivalent IQ.

## Key points

- Material-specific imaging adds a new dimension to fluoroscopic imaging.
- Tantalum-specific fluoroscopy offers higher image quality, with the same radiation exposure of conventional imaging.
- Tantalum-specific fluoroscopy requires lower radiation doses to achieve image quality comparable to conventional imaging.

## Background

The widely used detector dose-driven exposure control (DEC) in angiography is designed to maintain a constant detector dose, measured by the flat panel detector, in order to produce images of nearly constant noise level [1, 2]. DEC is not designed to consider the material of interest and does not differentiate between scattered and direct imaging x-rays, both contributing to the detector dose. Hence, there a limited means to optimise image quality (IQ) and radiation exposure for a specific diagnostic task [2, 3].

The recently introduced contrast-to-noise ratio (CNR)-driven exposure control (CEC) (OPTIQ, Siemens Healthineers, Forchheim, Germany) aims at reaching a given material-specific CNR in angiography [4–6]. CEC considers the object of interest with its material composition, size (“spatial frequency”) and average velocity. CEC estimates first the patient equivalent thickness in terms of water equivalent thickness (WET). Based on WET, CEC selects the image acquisition parameters precalculated using Monte Carlo simulation providing the requested CNR at the lowest possible incident air kerma at the interventional reference point (*K*_a,r_) rate [2, 3, 7].

Phantom experiments indicate a high skin dose reduction potential for materials with a high K-edge, as for example tantalum (Ta) [2, 3]. Alternatively, if improved visibility of tantalum-based embolisation material is requested by the interventional physician in order to increase the safety during the procedure, its visibility could be improved while keeping patient exposure stable [8]. A Ta-specific imaging approach could be of particular importance during the treatment of arteriovenous malformations, which are mostly present in younger patients. Embolisations of arteriovenous malformations are usually complicated and involve long fluoroscopy times [9, 10].

Up to date, it is unclear to which extent the results from the previously conducted phantom experiments with material-specific exposure control can be transferred into clinical practice [2, 3]. However, the comparison of different image acquisition protocols requires multiple acquisitions of the same image with different parameters, which is considered unethical and illegal in human volunteers or patients. Therefore, a realistic comparison can only be made in animal experiments.

The aim of this animal study was both, to compare the fluoroscopic IQ between Ta-specific CEC and conventional DEC, while maintaining the *K*_a,r_, and to compare the *K*_a,r_ while maintaining the IQ between Ta-specific CEC and DEC in abdominal angiography.

## Methods

### Animal experiment

The study was conducted in accordance with the European Directive 2010/63/EU and with the German law for animal protection (TierSchG). All experiments were approved by the local animal ethic committee (Lower Saxony State Office for Consumer Protection and Food Safety, LAVES 18/2809). Seven female domestic pigs weighing 68−88 kg were used. Anaesthesia was induced by intramuscular injection of 10 mg/kg tiletamine/zolazepam (Zoletil®, Virbac, Switzerland) and atropine (Atropinsulfat, BBraun, Germany) followed by intravenous injection of 10 mg/kg of propofol (Narcofol®, CP Pharma, Germany) to enable endotracheal intubation. Animals were maintained under general anaesthesia using an isoflurane precision vaporiser and mechanically ventilated (air-oxygen mixture 1:1; pIso > 1.8 mmHg). The breathing rate was set to 12 breaths per minute and a ventilation volume of 8−10 mL/kg per breath was chosen, based on the end-tidal CO_2_ (35−45 mmHg) concentration. The depth of anaesthesia was continuously monitored (ECG, capnography, body temperature, blood pressure, and O_2_ saturation). Animals received continuous fluid therapy (Ringer’s lactate, 10 nmg/kg/h), had a urinary catheter, and were positioned on a warming pad to maintain body temperature. Analgesia was achieved by an initial systemic intravenous dose of 4 mg/kg of carprofen (Rimadyl®, Zoetis, USA). Lidocaine (1 mg/kg, Xylocain, Aspen, Germany) was locally infiltrated at the arterial access sites. To obtain series of sharp images without movement of the diaphragm, the animals received an intravenous injection of 0.1 mg/kg of pancuronium (Pancuronium 2 mg/mL, Rotexmedica GmbH, Germany) every 1.5 to 2 h during anaesthesia. At the end of the experiment, the pigs were euthanised under deep anaesthesia by intravenous injection of 15–25 mL/animal of T61 (MSD, Unterschleißheim, Germany) until heart arrest was confirmed.

### Catheterisation

**Fig. 1:**
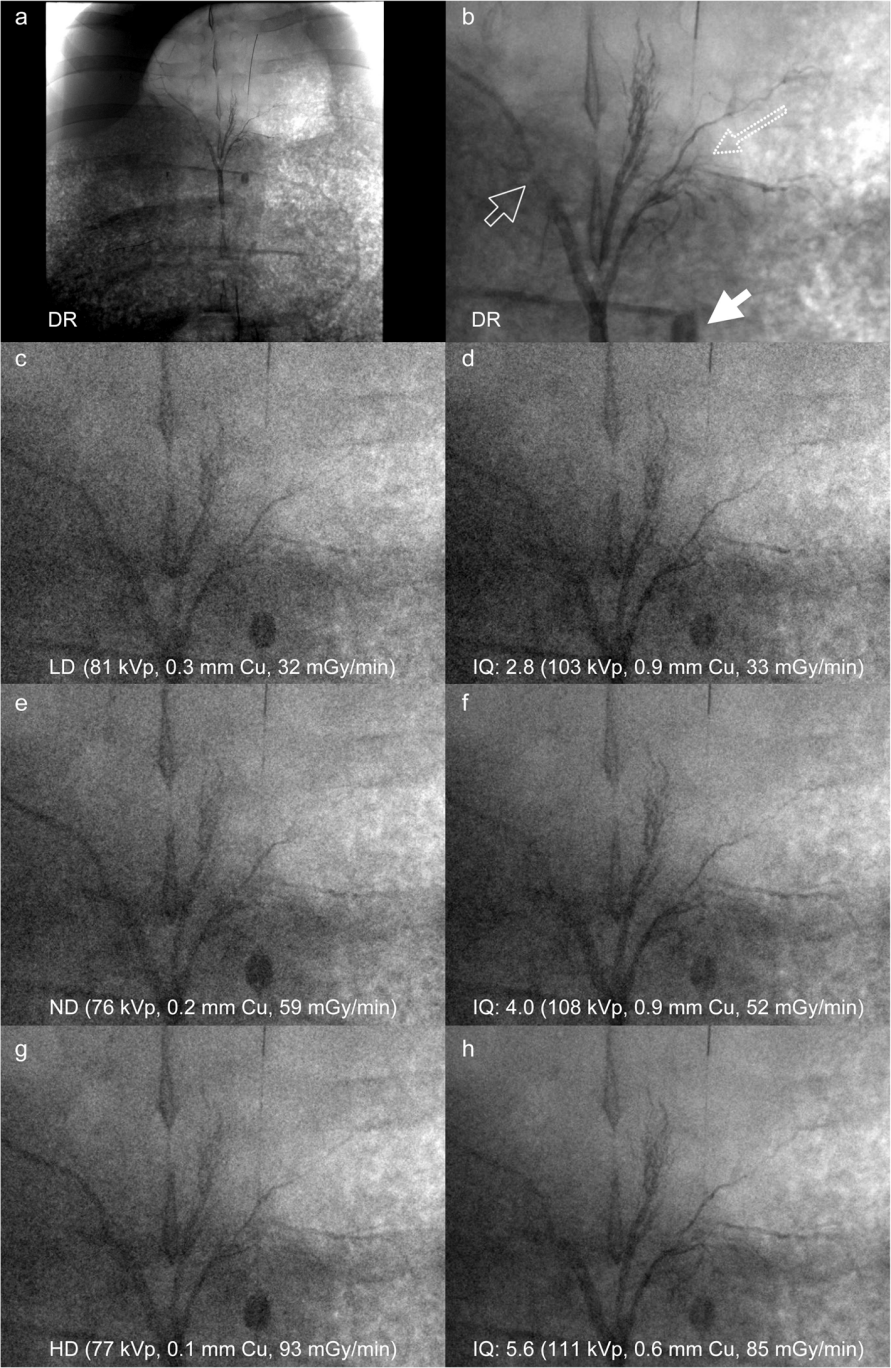
Example of fluoroscopic images of the liver at 32 cm water value acquired with the detector dose-driven exposure control using the three detector dose levels (LD low dose, ND normal dose, HD high dose) in comparison with fluoroscopic images acquired with the Ta-specific contrast-to-noise ratio-driven exposure control at a comparable incident air kerma rate (dose equivalent Ta image). The first row shows the overview image (**a**) and the magnified reference image (**b**), which were acquired with digital radiography (DR). The iodine mixed with cyanoacrylate-filled vessels and the iodine-filled balloon are indicated by the open and closed arrow. The dotted arrow indicates the tantalum filled gastric and liver arteries. *IQ* Image quality level

After local anaesthesia, a 5F introducer sheath was inserted into both femoral arteries under sonographic guidance. Appropriate angiographic catheters were then used to catherise the liver and the kidney. Embolisation of an appropriate subsegmental liver and kidney artery was conducted using tantalum-based embolisation material (Onyx®, Medtronic, Tolochenaz, France). In order to simulate iodine injections, an adjacent subsegmental liver and kidney artery occlusion was conducted with Lipiodol (Guerbet, Sulzbach, Germany) and cyanoacrylate glue (Histoacryl, B|Braun, Rubi, Spain) using a 4:1 ratio. A 3F Thru-Lumen embolectomy Fogarty catheter (Edwards Lifesciences Corp, Irvine, USA) filled with Iomeprol 300 (Bracco Imaging, Konstanz, Germany) was placed in the aorta holding a 0.0018” steel guidewire (Ashaee 18 guidewire, ASAHI INTECC CO LTD, Aichi Japan; V18^TM^ Control Wire, Boston Scientific, Ratingen, Deutschland). Furthermore, a 0.0014” micro guide wire (Tenor, Transcend, MeritMedical, Jordan UT, USA) within a microcatheter (Maestro, MeritMedical, Jordan, UT, USA) and 5F guide catheter was placed in a liver artery. Overall, nine imaging scenarios were created in the seven animals (Fig. 1).

### Image acquisition

In order to simulate different patient absorptions, doublets of polymethyl-methacrylate plates (30 × 30 × 2 cm^3^), combined with aluminium plates (30 × 30 × 0.2 cm^3^) were used to simulate 25 mm of soft and bone equivalent tissue. Two and four doublets were placed below the examination table using a custom-made holder and thus simulated additional 5 cm or 10 cm soft and bone equivalent tissue [2].

**Fig. 2:**
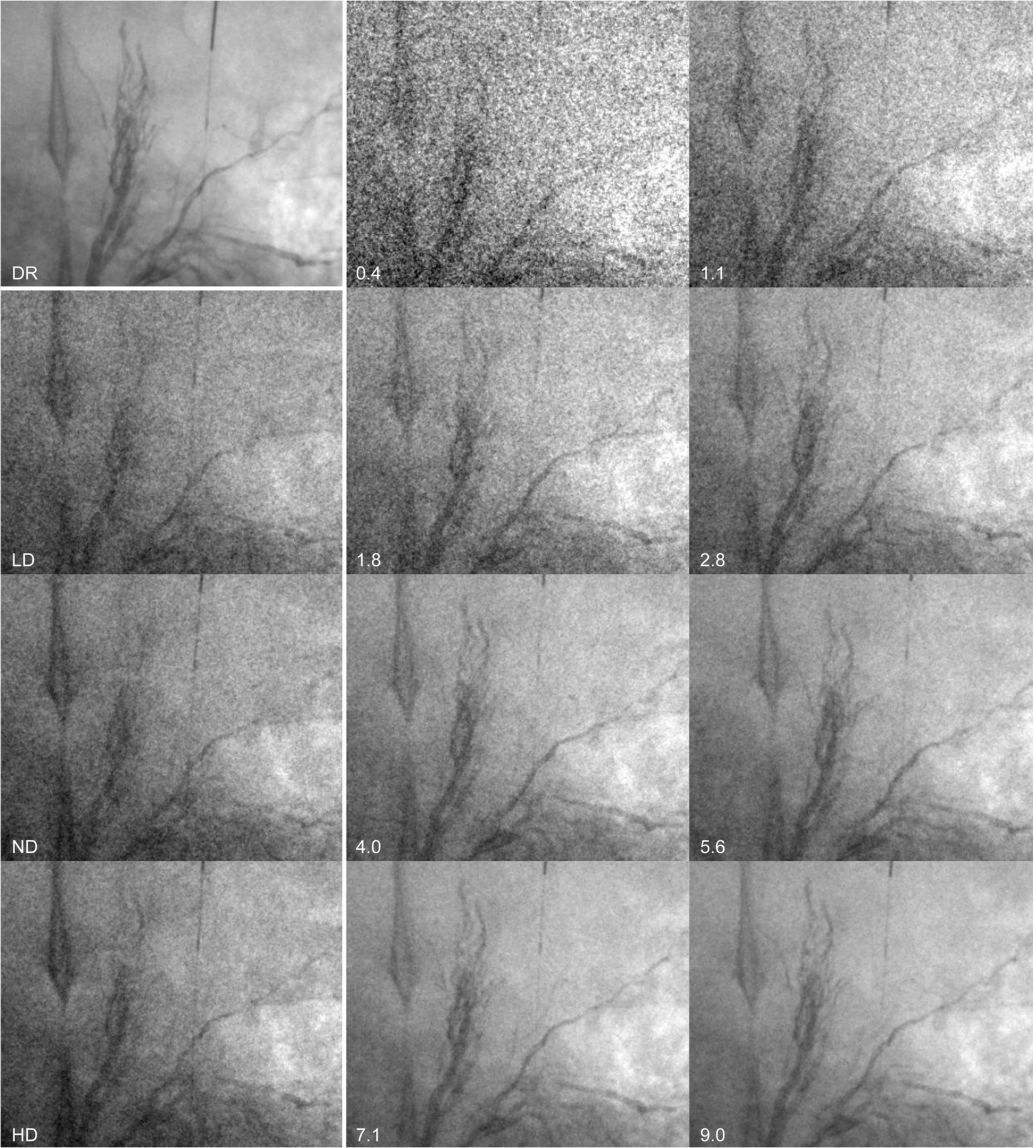
Images of Onyx® embolised liver and gastric arteries at 27 cm water equivalent thickness acquired with digital radiography (DR) as reference image, DEC using the three radiation dose levels (LD Low dose, ND Normal dose, HD High dose) and CEC using different image quality levels (IQ 0.4–9.0)

Imaging was conducted using a robotic C-arm angiography system (ARTIS pheno®, Siemens Healthcare GmbH, Forchheim, Germany), incorporating both types of exposure control (CEC and DEC). Fluoroscopic images of each scenario were acquired in one session using three clinically established DEC protocols (Table 1) with three different dose levels—low dose (LD); normal dose (ND); high dose (HD)—followed by the corresponding Ta-specific CEC protocols (Table 2). The acquisition of one scenario took about 4 to 6 min. Considering the different operating principle of CEC as compared to DEC, CEC was parametrised by different settings. CEC aims for a predefined material-specific, spatially and temporally frequency-dependent CNR instead of a constant detector dose at the image receptor. Image quality of CEC was parametrised by the following three parameters: the IQ level, which is proportional to CNR^2^; the IQ gradient; and the reference water value, at which the specified IQ level should be reached if IQ gradient is not 0. The IQ gradient allows adjusting the IQ dependent on the water equivalent thickness (WET), which is constant for an IQ gradient = 0 and increases strongest towards thinner regions for an IQ gradient = 1.0. In this study an IQ gradient = 0.5 was used, as this provides a nearly constant image noise [2]. The IQ levels were set in small increments to provide a close comparison of IQ and *K*_a,r_ of fluoroscopic images acquired using CEC with the fluoroscopic images acquired using DEC (Fig. 2). Scenes were acquired with the animal placed in the isocentre, a field of view of 42 cm, and a source to image distance of 110 cm. The acquisition time of one fluoroscopic scene covered approximately 75% of a breathing cycle. Due to the different acquisition characteristics of both exposure controls, the image acquisition protocols were first optimised to determine the appropriate image acquisition parameters. Finally, six imaging scenarios acquired with DEC and CEC were compared in this study.

**Table 1:** Fluoroscopic image acquisition protocols of the detector dose-driven exposure control

Parameter	Detector dose-driven exposure control		
Radiation dose level	Low dose	Normal dose	High dose
Acceleration potential plateau (kV)	81	73	70
Maximum pulse width (ms)	12.5	12.5	12.5
Minimum Cu-Filter (mm)	0.3	0.2	0.1
Maximum Cu-Filter (mm)	0.9	0.6	0.3
Focal spot	Small	Small	Small
Detector-dose (nGy/pulse)	45	55	65
Exposure point reduction	2	2 EP	0 EP
Maximum air kerma rate (mGy/min)	160	160	160
Frame rate (frames/s)	7.5	7.5	7.5

**Table 2:** Fluoroscopic image acquisition protocols of the Ta-specific contrast-to-noise ratio-driven exposure control

Parameter	Ta-specific contrast-to-noise ratio-driven exposure control
Spatial frequency (lp/mm)	1.4
Average velocity (mm/s)	15
Material of interest specific	tantalum
Image quality levels	0.4, 0.7, 0.9, 1.1, 1.3, 1.6, 1.8, 2.2, 2.5, 2.8, 3.2, 3.6, 4.0, 4.5, 5.0, 5.6, 6.3, 7.1, 9.0, 18.0
Image quality gradient	0.5
Maximum air kerma rate (mGy/min)	160
Minimum Cu filter (mm)	0.1
Maximum Cu filter (mm)	0.9
Frame rate (frames/s)	7.5
Reference water value (mm)	230

Postprocessing was minimised in order to reduce its possible effects and contained only a quadratic base curve and a gamma correction. All other post processing such as edge enhancement, noise reduction, or temporal averaging was switched off by using the service mode. The images were transferred for further evaluation to the Picture Archiving and Communication System. Images contained all of the image acquisition parameters, the WET of the projection, and the *K*_a,r_ measured using the dose-area product meter, which is incorporated into the x-ray source and the collimator assembly [11, 12].

### Image quality assessment

#### Dose equivalent imaging

First, the Ta-CEC acquired (dose equivalent Ta image) fluoroscopic image with an equivalent K_a,r_ compared to the DEC acquired fluoroscopic image (DEC-image) was selected. The dose equivalent Ta image was defined as the image with a *K*_a,r_ closest to the *K*_a,r_ of the DEC image and whose *K*_a,r_ was no greater than 5% above the *K*_a,r_ of the DEC image. The image dataset containing the LD, ND, or HD DEC image with the respective dose equivalent Ta image were presented on a workstation (Visage 7.1.15, Visage Imaging, Berlin, Germany) to three blinded readers with 5, 8, and 9 years of experience in angiography (Fig. 1). A digital radiograph of the respective Ta structure served as reference. The readers evaluated the IQ of the Ta structure using the five point Likert scale given in Table 3.

**Table 3:** Five-point Likert scale used for qualitative image assessment of tantalum structures

Score		Image quality
5	Excellent	“More than diagnostic” to good image quality
4	Good	Good image quality
3	Sufficient	Sufficient image quality
2	Partial diagnostic	Partially insufficient image quality
1	Non-diagnostic	Insufficient image quality

#### Image quality equivalent imaging

In a second step, the fluoroscopic images acquired with Ta-CEC and different IQ levels were compared by the readers to the fluoroscopic images acquired with DEC and different dose levels. The readers were asked to select the fluoroscopic image acquired with Ta-CEC that most closely provided the IQ acquired with DEC (Ta-IQ equivalent image). For each image dataset, the radiologist specifically focused on the depiction of the tantalum structures. The *K*_a,r_ of the Ta-IQ equivalent image was subsequently documented and compared to the *K*_a,r_ of the corresponding DEC image (Fig. 2).

### Statistics

Descriptive statistical analyses were calculated (mean value ± standard deviation) [13–15]. Interobserver agreement between the three readers was calculated using the two-way random intraclass correlation coefficient with absolute agreement. The following classification was used for interpreting the agreement: poor (< 0.40); fair (0.40−0.59); good (0.60−0.74); excellent (≥ 0.75) [16]. The comparison of IQ ratings of the DEC image with IQ rating of the corresponding dose equivalent Ta image was performed using the visual grading characteristics (VGC) analyser software [17]. In VGC analysis, IQ ratings for two different conditions are compared by producing a VGC curve, similar to how the ratings for normal and abnormal cases in receiver operating characteristic analysis are used to create its curve. The software computes the area under the visual grading curve (AUC_VGC_) with 95% confidence interval using bootstrapping (*n* = 2,000), taking possible dependencies between ratings into account. An AUC_VGC_ value of 0.5 reflected similar IQ for the two exposure controls, while an AUC_VGC_ > 0.5 indicated superior IQ, and an AUC_VGC_ < 0.5 indicated inferior IQ produced by CEC.

Possible *K*_a,r_ differences between the DEC-image and the dose equivalent Ta image were assessed via paired *t* test according to Norman [13] who demonstrated the robustness of parametric tests. To assess the dose differences between the DEC image and the respective Ta-IQ equivalent image, the mean of the *K*_a,r_ values selected by the three readers was first calculated and then compared with the paired *t* test. Normal distribution of the dose data was confirmed level-wise using the Shapiro-Wilk test. Differences in *K*_a,r_ reduction between the different dose levels were assessed with the ANOVA test and post-hoc paired *t* test with Bonferroni correction. A *p* value < 0.05 indicated significance.

The required sample size was conservatively estimated, based on the estimated dose difference of at least 30 ± 20% with an abnormal distribution of the paired acquisitions. This requires at least six scenarios to achieve a power of 0.8 at an α error of 0.05 [18]. The statistical analyses in addition to the VGC software were performed with R (R version 3.6.3, http://www.r-project.org with package “IRR” version 0.84.1) and the sample size estimation with G*Power (G*Power3.1.9.2, Kiel, Germany).

## Results

### Dose equivalent imaging

**Fig. 3:**
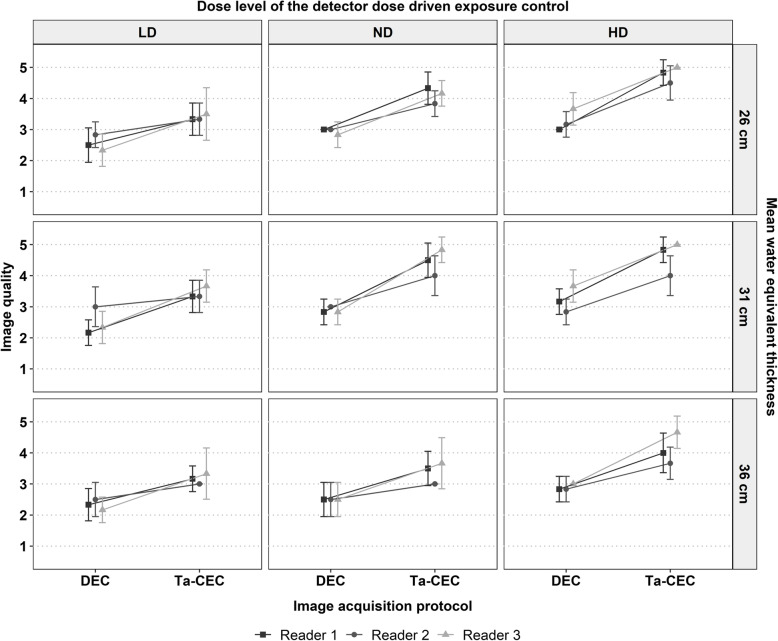
Average image quality ratings of the three readers for the detector dose-driven exposure control (DEC) and the dose equivalent Ta image acquired with the contrast-to-noise ratio driven exposure control (Ta-CEC), dependent on the dose level of the detector dose-driven exposure control and the water equivalent thickness

Mean WET of the six fluoroscopic scenarios was estimated by the angiography system as follows: low WET (25.8 ± 1.1 cm, mean ± standard deviation); medium WET (30.9 ± 0.8 cm); and high WET (36.4 ± 1.0 cm). The interobserver agreement for the assessment of the IQ of DEC (0.43) and Ta-CEC (0.56) was fair. Every reader observed a substantial increase in IQ for the dose equivalent Ta image as compared to the DEC image as demonstrated in Fig. 3 and Table 4. Overall, the average IQ of the dose equivalent Ta images (3.9 ± 0.8) was significantly higher as compared to the corresponding DEC images (2.8 ± 0.5, mean ± standard deviation), with a small but significantly lower *K*_a,r_ (52.4 ± 37.1 mGy/min *versus* 54.6 ± 38.5, *p* < 0.001). Regarding the radiation dose levels of DEC, the highest average increase in IQ in the dose equivalent Ta images was observed for the HD level (3.1 ± 0.5 *versus* 4.5 ± 0.6), followed by the ND level (2.8 ± 0.4 *versus* 4.0 ± 0.7) and LD level (2.5 ± 0.5 *versus* 3.3 ± 0.5). Considering the change in average IQ dependent on WET, the increase in IQ of the dose equivalent Ta image was similar for the low (2.9 ± 0.5 *versus* 4.1 ± 0.8) and medium WET (2.9 ± 0.6 *versus* 4.2 ± 0.8), followed by the highest WET (2.6 ± 0.5 *versus* 3.6 ± 0.7). The average IQ of the dose equivalent Ta image dependent on WET and DEC dose level was significantly higher as compared to the DEC image (Table 4).

**Table 4:** Incident air kerma rate at the interventional reference point and average image quality of all three readers of fluoroscopic images acquired with detector dose-driven exposure control (DEC) and Ta-specific contrast-to-noise ratio-driven exposure control (CEC), while maintaining the incident air kerma rate

Mean WET (cm)	Dose level (DEC)	Incident air kerma rate at the interventional reference point			Average image quality			
		DEC	CEC	***p*** value	DEC	CEC	AUC	***p*** value
26	LD	6.6 ± 1.4	6.2 ± 1.3	0.003	2.6 ± 0.7	3.4 ± 0.7	0.82 [0.68–0.93]	0.018
	ND	15.8 ± 3.9	15.4 ± 3.8	0.216	2.9 ± 0.4	4.2 ± 0.5	0.97 [0.89–1.00]	0.014
	HD	28.8 ± 7.6	28.5 ± 7.5	0.147	3.3 ± 0.6	4.8 ± 0.4	0.98 [0.91–1.00]	0.017
31	LD	24.5 ± 5.1	23.5 ± 6.3	0.165	2.4 ± 0.8	3.3 ± 0.6	0.84 [0.64–1.00]	0.020
	ND	53.0 ± 4.4	50.8 ± 3.4	0.137	2.8 ± 0.5	4.4 ± 0.6	0.97 [0.89–1.00]	0.022
	HD	86.1 ± 4.6	81.0 ± 7.7	0.044	3.2 ± 0.6	4.6 ± 0.6	0.93 [0.79–1.00]	0.014
36	LD	60.7 ± 4.5	58.1 ± 7.1	0.182	2.1 ± 0.7	2.9 ± 0.8	0.85 [0.67–1.00]	0.019
	ND	86.4 ± 5.7	83.7 ± 6.7	0.163	2.4 ± 0.6	3.3 ± 0.7	0.85 [0.68–0.93]	0.011
	HD	129.4 ± 8.4	124.2 ± 14.1	0.212	2.8 ± 0.5	3.9 ± 0.7	0.93 [0.79–1.00]	0.014

Values are given as mean ± standard deviation. *AUC* Area under curve, *CEC* Contrast-to-noise ratio-driven exposure control, *DEC* Detector dose driven exposure control, *LD* Low dose, *ND* Normal dose, *HD* High dose, *WET* Water equivalent thickness

### Image quality equivalent imaging

**Fig. 4:**
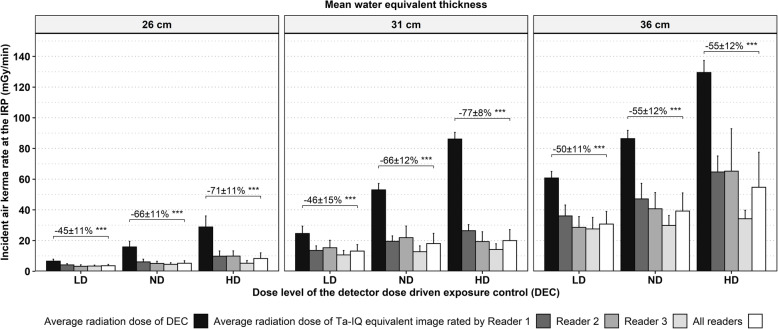
Incident air kerma at the interventional reference point (IRP) of fluoroscopic images acquired with detector dose driven exposure control (DEC) at three dose levels (LD: Low dose, ND: Normal dose, HD: High dose) and three water equivalent thicknesses, compared to the IRP of the image acquired with Ta-specific contrast-to-noise ratio driven exposure control at comparable image quality of the Ta structures. ***p < 0.001. *IRP* Interventional reference point

The assessment of *K*_a,r_ of the Ta-IQ equivalent images demonstrated a good interobserver agreement (0.73). Overall, the average *K*_a,r_ of the Ta-IQ equivalent images was 59 ± 16% (mean ± standard deviation) *versus* lower (*p* < 0.001) as compared to the *K*_a,r_ of the DEC images. Regarding the radiation dose levels of DEC, the highest average *K*_a,r_ reduction of the Ta- IQ equivalent images was documented for the HD level (68 ± 15%), followed by the ND (62 ± 13%) and LD level (47 ± 13%). Considering the dose difference dependent on the WET, comparable *K*_a,r_ reductions of the Ta-IQ equivalent image were observed for the low (61 ± 13%) and medium WET (63 ± 15%), while K_a,r_ reduction for the highest WET (54 ± 7%) was lower. For each WET and DEC dose level, significant averaged *K*_a,r_ reductions were noted (*p* < 0.001, Fig. 4). In detail, the Ta-IQ equivalent images selected by each reader provided the same IQ as compared to the DEC image at significantly (*p* ≤ 0.011) lower *K*_a,r_ for every WET and DEC dose level. Average *K*_a,r_ reductions increased significantly from the LD to the ND level for each WET (*p* ≤ 0.014) and were comparable for the low and medium WET and lower for the high WET. Between the ND and HD level a significant average *K*_a,r_ reduction was only noted for them medium WET (*p* ≤ 0.007).

## Discussion

The results of this animal study demonstrate that a tantalum-specific contrast-to-noise ratio-driven exposure control (Ta-specific CEC) significantly improves the depiction of tantalum as compared to conventional DEC in abdominal angiography maintaining a constant *K*_a,r_. Alternatively, the skin radiation dose can be reduced significantly with Ta-specific CEC maintaining the IQ of Ta. The effective skin dose reduction is beneficial, in particular in long lasting procedures in young patients using tantalum based embolisation material in arteriovenous malformations. In addition, IQ can be adjusted over a much wider range using CEC as compared to DEC, as indicated in Fig. 2 and Table 4.

The results confirm the results of the recently conducted phantom studies by Dehairs et al. [2] and Werncke et al. [3], who reported a significant reduction in skin radiation dose by using Ta-specific CEC instead of DEC in cardiac and abdominal angiography imaging. As expected from these studies, the highest dose *K*_a,r_ reductions were noted for the low and medium WET. The increase of the *K*_a,r_ reductions with the dose level underlines the importance of material-specific imaging. It also indicates that DEC is not optimised for the imaging of tantalum and can lead to unnecessary high-dose levels.

Our study augments the results of a clinical study by Vogl et al. on 106 patients receiving transarterial chemoembolisation using ARTIS pheno® (Siemens Healthineers, Forchheim Germany), which reported superior IQ in fluoroscopy at a lower radiation dose as compared to the predecessor using DEC, as higher copper filtration was applied [19]. However, their study compared the IQ and dose between two systems under non-identical conditions and focused on the imaging of iodine and iron, which limits the potential of skin radiation dose savings as the material-specific advantages of tantalum were not investigated. The main advantage of this animal study was that multiple identical acquisitions of the same scene with different absorption thicknesses could be acquired.

The skin radiation dose reductions observed in our study can be explained by the different principle of CEC as compared to DEC. DEC aims to achieve a predefined radiation dose at the image receptor, which is achieved by an algorithm that does not take the material properties and acquisition settings into account [20]. CEC, which considers the acquisition settings, object and material properties, adjusts the x-ray spectra in order to optimise the absorption difference between the material-specific properties of the object of interest and the soft tissue, while the entrance skin dose is minimised. According to the K-edges of the materials of interest, CEC varies the tube voltage and copper filtration to a much larger extent than DEC [2, 3]. The absorption of Ta with a K-edge of 67.4 keV, using Ta-CEC is substantially increased with the higher tube voltage as compared to DEC, which is optimised for the depiction of lighter materials as iodine (K-edge 33 keV) and iron (K-edge 7.1 keV) [21]. The simultaneous use of thicker copper filters reduces the low-energy x-rays, which are mainly absorbed by soft tissue and contribute to a lower fraction to the depiction of tantalum. Therefore, the skin radiation dose applied by Ta-CEC can be significantly lowered as compared to DEC, while the CNR of Ta is maintained [2]. A possible drawback of the Ta-specific imaging can be the simultaneously reduced contrast of light materials, *e.g.*, iodine, iron and bony structures. The reduced visibility of the light materials simulated by the 0.018 in guidewire, the iodine-filled Fogarty balloon, and the Lipiodol/cyanoacrylate-filled vessel in Fig. 2 can be compromising. On the other hand, this effect can be beneficial as the bony structures become more transparent. However, in embolising procedures, the embolic agent is in the focus of interest and if clear depiction of other materials is desired, the appropriate material-specific protocol can be chosen.

The newly introduced parameter “average velocity” in CEC, which limits the pulse width, has an important impact on the possible skin dose savings, as it is directly linked to the x-ray tube capacity, which limits the selection of acquisition settings. For this reason, the slower the object moves, the greater the potential skin dose savings. In this study, we used an average velocity of 15 mm/s, which is used in clinical practice for the depiction of guide wires in abdominal interventions. In regions with very little respiratory motion, such as the pelvis, a lower “average velocity” and thus a lower restriction on maximum pulse widths is possible, which can result in even higher skin dose savings, *e.g.*, for uterine or prostate artery embolisation. The IQ gradient of 0.5 was selected as it provides a similar image noise between the different IQ levels. In clinical routine, a constant IQ with an IQ gradient of 0.0 would be preferred in order to fulfil the “as low as reasonably achievable”, ALARA, principle.

Several limitations of our study need to be acknowledged. Firstly, only a small subset of Ta-CEC acquisition protocols could be compared to the conventional DEC protocols. As already described, the new parameters “average velocity” and to a smaller extend “spatial frequency”, which limit the focal spot, have a direct impact on the selected x-ray parameters by the CEC and thereby limit the possible radiation dose savings [2]. Therefore, both parameters—and also IQ—should be tailored to the diagnostic task and body region. Secondly, the skin entrance dose was not directly measured. Instead, the study analysed *K*_a_,_r_, measured by the DAP ionisation chamber. The advantage of this approach is that *K*_a_,_r_ is provided by International Electrotechnical Commission compliant angiography systems [12] and can easily be compared between different systems. Furthermore, the skin dose can be estimated based on the *K*_a_,_r_ [22]. Thirdly, image postprocessing was minimised to exclude its effect on IQ. Therefore, its effect on the patient dose was not investigated, although postprocessing is an important instrument of radiation dose reduction [23–26]. Finally, the effect of Ta-CEC on digital angiography as compared to DEC was not evaluated due to several reasons. Extensive acquisitions of digital radiography, such as those performed for fluoroscopy in this study, would require many respiratory arrests and would cause significant stress to the animal. According to phantom experiments, we expect that the influence of tantalum-specific imaging on digital radiography is comparable to its influence on fluoroscopy. Furthermore, fluoroscopy of Ta containing embolisation material is regularly used in clinical practice.

In conclusion, Ta-CEC significantly improves the fluoroscopic depiction of Ta maintaining a constant incident *K*_a,r_ when compared to DEC. Alternatively, the *K*_a,r_ can be significantly reduced by using Ta-CEC instead of DEC, if a comparable depiction of tantalum is warranted. Thus, the use of Ta-CEC allowed for a significant improvement in IQ or dose reduction in fluoroscopic imaging of Ta containing embolics, which are regularly used in embolisation procedures in young patients.

## Data Availability

The datasets analysed in the present study are available from the corresponding author on reasonable request.

## Abbreviations

AUC_VGC_: Area under the visual grading curve
CEC: Contrast-to-noise ratio-driven exposure control
CNR: Contrast-to-noise ratio
DEC: Detector dose-driven exposure control
HD: High dose
IQ: Image quality
*K*_a,r_: Incident air kerma at the interventional reference point
LD: Low dose
ND: Normal dose
Ta-CEC: Tantalum-specific CEC
Ta-IQ: Tantalum-specific IQ
VGC: Visual grading characteristics
WET: Water equivalent thickness
